# Enhancing Security of Web-Based IoT Services via XSS Vulnerability Detection †

**DOI:** 10.3390/s23239407

**Published:** 2023-11-25

**Authors:** Jemin Kim, Joonseok Park

**Affiliations:** Department of Electrical and Computer Engineering, Inha University, Incheon 22212, Republic of Korea; kimjemin@inha.edu

**Keywords:** Internet of Things (IoT), application layer, web application security, Cross-Site Scripting (XSS), dynamic taint analysis, concolic execution

## Abstract

The Internet of Things (IoT) technology is experiencing significant growth and integration into various aspects of daily life. With the rising number of connected devices, diverse security challenges are emerging as substantial threats to IoT. Cross-Site Scripting (XSS) is one of the major security risks in web services and so is within the application layer of IoT. Many existing web applications remain susceptible to XSS vulnerabilities. In this paper, we propose an XSS detection scheme aimed at enhancing the security of IoT, particularly concerning web application services. To achieve this, we developed a framework for combining symbolic execution and dynamic taint analysis to provide a comprehensive security assessment. Our objective is to increase the ratio of vulnerability detection while avoiding false alarms and keeping the required analysis time as minimal. To realize our idea, we have defined an instrumentation scheme for taint analysis and concolic executions and automated the process of vulnerability detection for a web application. Our framework is capable of pinpointing the precise locations of security vulnerabilities and the exact input datasets at risk of XSS threats. Subsequently, the detected flaws can be easily removed. The experimental results demonstrate the validity of the proposed scheme. We achieved a detection rate of XSS threats of 90.62% using a test set of SecuriBench Micro and 69.11% using OWASP while showing 0% false positives.

## 1. Introduction

**Figure 1:**
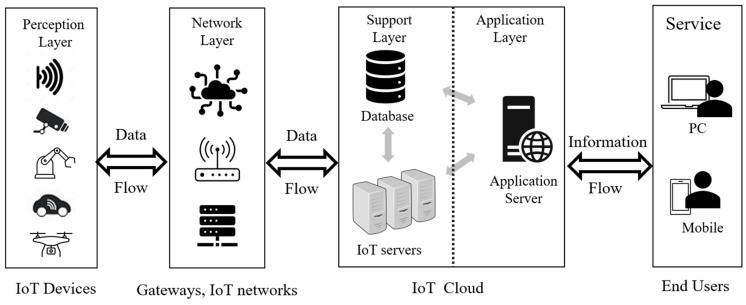
Components of four architectural layers of IoT.

The Internet of Things (IoT) has dramatically changed human interactions with physical environments by connecting a multitude of devices and sensors via the internet. The primary objective of IoT is to autonomously collect and transfer data with minimal human interventions. This technology is used in many application-domains, including the supply chain, transportation, logistics, automation, healthcare, and diverse CPS (Cyber Physical Systems) [1]. As the number of devices connected to IoT increases, security becomes one of the most critical issues in IoT development. Security requirements are essential in the context of IoT to maintain trust among clients. To implement a secure IoT, many technical concerns are required. Because of the heterogeneity of connected devices and the diversities of computing resources and software components, IoT is vulnerable to diverse security threats. Figure 1 shows the components of four different architecture layers of IoT and the data and information flows between the components, and Table 1 provides a summary of those layers and their representative security attacks [2].

The perception layer, also known as a sensor layer or device layer, is composed of small low-power sensor nodes. It identifies things and is responsible for collecting data using many different types of sensors, actuators, and edge devices. It is primarily used for environmental monitoring, data collection, and communication purposes. The network layer carries and transmits data from the perception layer to further computational units. These sensor networks utilize wired or wireless communication. The network layer operates similarly to traditional TCP/IP; therefore, it has similar security issues. The IoT support layer manages various services, resources, and functionalities that provide essential support for the operations of IoT systems. It plays a foundational role in ensuring the reliability, scalability, and overall efficiency of IoT deployment. The application layer represents the top layer of the IoT framework and is responsible for delivering value and functionality to end-users, businesses, and various applications. It is where data, generated by IoT devices and sensors, are processed, analyzed, and transformed into meaningful information or actions [2,3].

Among the four layers, the application layer directly deals with end-users; hence, privacy and data protections are major concerns at this layer. The functions of the application layer encompass data processing/analysis, diverse application logics, data storage, user interfaces, and security/access controls. The user interfaces are usually implemented in web applications, mobile apps, or other means of access [4].

In this paper, we focus on the security enhancement of the application layer of IoT. One of the major security threats is Cross-Site Scripting (XSS attack). XSS occurs if malicious script input is injected into the generated HTML page. The prevention of XSS vulnerability is important because cookies, session data, and other sensitive data can be accessed by the XSS attacker, which can put the whole IoT at risk. Some of the modern web frameworks used to build IoT services provide ways to prevent or mitigate the potential risk of XSS [5,6]; however, legacy web frameworks do not offer such capabilities and remain vulnerable to XSS threats. In fact, over 53,000 sites continue to use legacy Java EE as a web framework [7]. Many smart IoT devices, such as smart cameras and wireless routers, allow device control via web browsers as well.

XSS attacks cause problems not only in the web application layer of IoT but also in conventional web services. However, XSS in IoT systems can have more serious consequences for two reasons. First, through reflected XSS, it is possible that an attacker gains access to cookies, session data, and privileges, and then compromises all physical devices within the IoT system. This poses a more significant threat, leading to issues in the physical system, for example, car accidents in ITS (Intelligent Traffic System), extending beyond the scope of attacks on typical web applications. Second, the IoT system is more vulnerable to persistent XSS attack, which means an attacker inserts malicious scripts into the servers’ storage. In IoT systems, data are collected from sensors and stored in the IoT database via the device management web interface. Due to constraints on computing resources and the security vulnerabilities in firmware, sensor devices are relatively more susceptible to security risks compared to regular web services. As listed in Table 1, the perception layer, composed of sensor devices, is susceptible to malicious code injection attacks. If illicit code resulting from an attack is stored in the IoT database, it may manifest as an XSS vulnerability in the application layer.

We present a novel approach for uncovering XSS vulnerabilities in legacy Java EE web applications: a dynamic taint analysis with concolic execution. Taint analysis examines the information flow from untrusted user input to malicious action to the system. Concolic execution, also known as concolic testing, is a software verification technique that systematically integrates symbolic execution and concrete execution [8]. Symbolic execution, which treats program variables as symbolic expressions, is used in conjunction with an automated theorem prover (constraint solver) to generate inputs for new test cases. The program under test is iteratively executed using these newly generated inputs until the test coverage reaches a satisfactory level. Throughout these concrete executions, dynamic analysis is performed. We implement this scheme as a framework in which taint analysis is recurrently executed until all reachable execution paths are examined. In each iteration of dynamic analysis, it identifies XSS risks based on provided inputs and ensuing control flows. Simultaneously, all execution path conditions are annotated, yielding corresponding symbolic expressions. These symbolic expressions are utilized by the constraint solver to generate new input sets, thereby enabling the execution of previously unexplored control flows. The method we propose is applicable not only to IoT systems but also to conventional web application servers.

The contributions of this research are three-fold. First, we propose a security-enhancing scheme for IoT application layers using XSS vulnerability detections on web applications. Second, we defined and automated an instrumentation scheme for concolic executions of a dynamic taint analysis scheme without false alarms while mitigating execution explosion via concolic executions. Lastly, our proposed scheme enables the automatic tracing of specific inputs that pose XSS threats and detects the vulnerable point of the web application.

A preliminary version of this paper has been presented at the International Conference on Next Generation Computing (ICNGC) 2022 and has been extensively revised and improved with results using enhanced implementation [9]. We have improved the detection ratio by enhancing the information flow analysis and widened applications focusing on IoT. We have strengthened our hypothesis by enhancing our detection methodology and comparing our achieved performance with other related works. We have improved our proposed framework by automating taint analysis and concolic execution procedures.

The remainder of this paper is as follows. Section 2 and Section 3 contain related research and preliminary explanations of the important literature for our work, respectively. Section 4 explains the SW components of our proposed scheme and the information flow analysis for integrating taint analysis and concolic execution. In Section 5, we present experimental results which show the validity of our proposed scheme. In Section 6, we conclude and discuss the current limitations of the proposed method and future research directions.

## 2. Related Works

Recently, there have been many research efforts to enhance the security of the IoT application layer by detecting XSS threats. Chaudhary et al. [10] propose a mechanism for protecting IoT from malicious XSS attacks targeting the smart devices that constitute the IoT. They compare requested script with XSS attacking script, which is stored beforehand, using the Boyer–Moore string matching algorithm. Chaudhary et al. [11] presented a method for detecting devices exposed to XSS by comparing injected strings targeting smart devices with a stored blacklist and exploring them via filtering. Boppana and Bagade [12] demonstrated the feasibility of attacking user sessions in a web application for monitoring an industrial IoT system and controlling the system on behalf of the actual user by launching an XSS attack using the unencrypted MQTT (Message Queue Telemetry Transport) protocol. By demonstrating this security threat, they raised concerns about the crucial need to devise strategies for large-scale IoT systems to defend against malicious attacks.

Numerous studies have explored the identification of XSS vulnerabilities and SQL injection attacks [13,14,15]. Among these, static taint analysis offers distinct advantages, such as analysis speed, no requirement for source code modifications, and upholding soundness [16,17,18]. Nonetheless, the mitigation of false positives remains a formidable challenge. In this context, TAJ [19] conducted various taint analysis assessments of large-scale Java Platform Enterprise Edition (JEE) applications. Their focused vulnerabilities were SQL Injection and XSS. They systematically applied several static taint analyses, and subsequently compared evaluations of the quantity of vulnerability detections (true positives and false positives) and the computational time requirements for each method. SpotBugs, the successor to FindBugs [20], is a widely used free open-source static analysis tool [21]. It is utilized not only for security checks but also extensively to enhance code quality. It examines Java applications at the bytecode level to detect various types of errors and bug patterns as well as potential performance issues. For security checks, SpotBugs offers FBwFindSecBugs (Find Security Bugs), a plugin dedicated to security audits of Java web applications. It handles XSS, SQL, and sensitive information exposure, helping developers easily identify and address security-sensitive areas.

Scriptguard [22] is a dynamic taint analysis framework which is designed for runtime auto-correction. The auto-correction is achieved via two distinct phases. First, it traces the execution path using the initial test input and records the problematic sanitization sequence. Next, it instruments the code for the appropriate sanitization method to handle identified problematic sanitization sequences on the execution path. It has similarities with our approach because it traces the execution path and instruments the code for the correct sanitization method. A grammar-based test input generation which automatically generates test inputs out of the given web page is presented in [23]. It enables automatic unit tests for XSS vulnerability. These studies have similarities with ours in a sense that they automate the dynamic taint analysis. However, the schemes presented in [22,23] lack consideration of the coverage of the analysis. Our proposed scheme aims to uncover all XSS vulnerability feasible execution paths. OWASP ZAP [24] is created by the OWASP foundation and is the most widely used free and open-source web application scanner. OWASP ZAP crawls and performs attacks to find vulnerabilities. It may suffer from the execution time explosion issue, which is common in dynamic analysis schemes.

There is much research on concolic execution. CUTE [8], DART [25], and KLEE [26] are the frameworks for concolic execution targeting C programs. jCute [27] and JDart [28] are targeted for Java programs. Some of the research on concolic execution has been adapted for taint analysis. TaintKLEE [29] shows the possibility of performing taint analysis in a C program. However, it only presented the analysis scheme, without the demonstration of a specific application of taint analysis. Jaint [30] presents a taint analysis method using the virtual machine extension function of Java PathFinder [31] and Jdart that can designate a taint source and a taint sink. In Jaint, the user has to write the description of the taint source and sink using the domain-specific language. In contrast, those description efforts by the user are relieved in our proposed system.

## 3. Preliminaries

### 3.1. Concolic Executions

Concolic execution traces the path while executing the initial input data concretely, and later uses symbolic execution to generate new input combinations based on the program’s conditional statements and control flow. It involves analyzing the source code to generate test inputs iteratively, enabling the exploration of feasible execution paths of the program. By iteratively generating test inputs and executing the software, this testing approach aims to reach all execution paths within the program.

**Figure 2:**
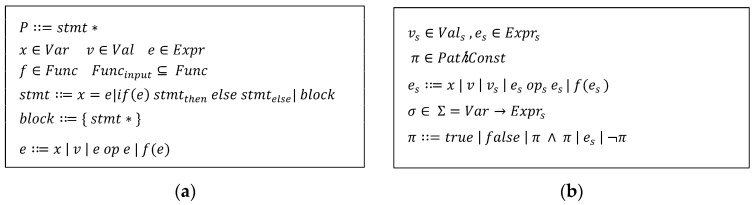
(**a**) Syntax definition of simple programming language and (**b**) extended grammar for symbolic execution.

In Figure 2a, the grammar program consists of stmts which can be derived to the assignment statement (x=e), if-structures ($if\left(e\right)\;{stmt}_{then}\;else\;{stmt}_{else}$), or block. Expression derives one of {variable(*x*), value(*v*), operation of values, function calls}. In Figure 1b, we expand upon the grammar production rules to accommodate symbolic executions. Symbolic execution represents program inputs and variables using symbolic values instead of concrete values. In symbolic domains, $v_{s}\in {Val}_{s}$ (symbolic value) and $e_{s}\in {Expr}_{s}$ (symbolic expression) are defined in the syntax. The mapping between variables to the symbolic value or expressions are defined as the symbolic state σ. Additionally, we introduce the syntactic domain π∈PathConst, denoting path constraints, which characterizes the executed control flow. The path constraint *π* is a logically conjunctive list of Boolean symbolic expressions.

Throughout the process of the symbolic execution of program P, each statement (stmt) in the program updates the symbolic value of variables, symbolic expressions, the symbolic state σ, and/or the path constraint *π*. When an assignment statement undergoes symbolic execution, the mapping in the symbolic state of the left-hand-side variable is updated to that of the right-hand side. The path constraint is updated only when a branch statement, such as if and if else statements, is encountered. If the condition in a branch statement evaluates to be true, the symbolically evaluated expression of the condition is conjuncted with *π*. Conversely, if the condition evaluates to be false, the negated symbolic expression of the condition is appended to *π*.

However, symbolic execution can suffer from the path explosion problem because it relies on executing all feasible execution paths. This can lead to a rapid increase in the number of paths to explore. Moreover, since it substitutes input values with symbols during execution, it may introduce inaccuracies in behavior when compared to actual execution. Another notable limitation arises when addressing external library calls that are hard to track symbolically. Concolic execution mitigates these shortcomings by using concrete values rather than symbolic ones in such scenarios. This approach also contributes to alleviating the path explosion problems associated with symbolic execution [32].

**Figure 3:**
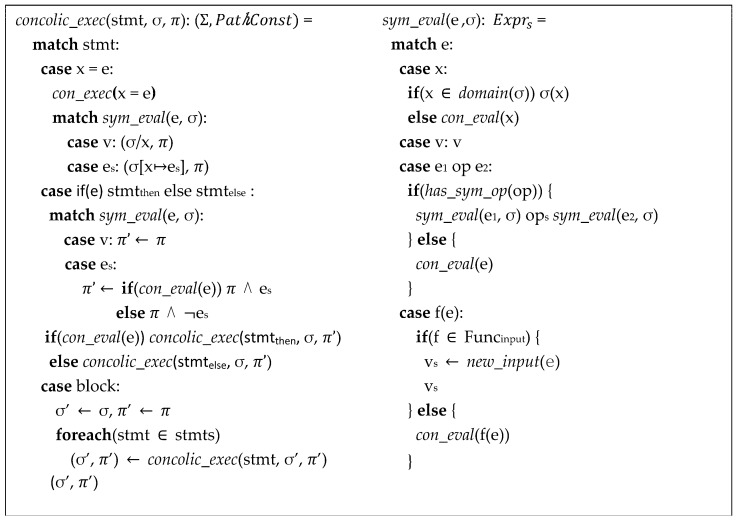
Semantics of concolic execution that is extended from the symbolic evaluation.

Similar to the symbolic executions, in each concolic execution of stmt, it updates the symbolic value of variables, symbolic expressions, the symbolic state σ, and the path constraint *π* as shown in Figure 3. The only difference from the pure symbolic execution is that if an expression is evaluated as a concrete value, which is derived from the concrete input value, the concrete value of x is excluded from the symbolic state σ (σ/x). Otherwise, it updates the symbolic state σ by [x↦e_s_]. When it comes to an if statement, if the conditional expression, e, can be evaluated to a concrete value, then π remains unchanged. However, if the condition e is a symbolic expression, it is conjoined with π. If the condition e evaluates to be true, the then part is concolically executed. If it evaluates to be false, the else part is concolically executed. For a block of code, the individual statements within the block are executed in sequential order.

In sym_eval (symbolically evaluating), a concrete expression is translated into a symbolic expression. By contrast, con_eval generates a concrete value by real execution of the given expression and/or functions. For variables x, if a symbolic value, v_s_, can be obtained from the symbolic state σ, which means σ(x) = v_s_, vs is returned; otherwise, a symbolic expression is generated using the concrete value by using con_eval. For operations, if there exists a symbolic operator (has_sym_op), a new symbolic expression is created by concatenating the left operand, symbolic operator, and right operand expressions. Otherwise, it returns the actual evaluated concrete value (con_eval). For function calls, if the function is an input handling function, a new symbolic value is generated and returned (new_input). Otherwise, if it is an external library function call, the concrete value, which is an actual result of the function, is returned.

### 3.2. Dynamic Taint Analysis

Taint analysis is the process of identifying risky flows within a program, particularly those that involve sensitive information, originating from untrusted external inputs [6]. Dynamic taint analysis involves analyzing the data flow from user inputs, which can potentially be the cause of vulnerabilities, to the execution of functions where vulnerabilities may occur while the program is running. In this research, we combine concolic execution and dynamic taint analysis to perform XSS vulnerability detection.

**Figure 4:**
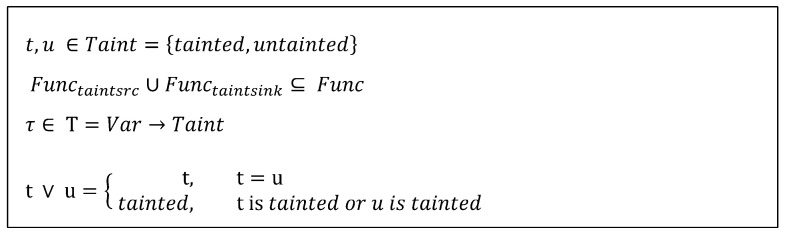
Syntax definition of taintedness, taint state, and taint join operation.

As in Figure 4, when a variable is returned from a function that can potentially be the source of a vulnerability, the variable is considered tainted, and any variables that have data propagated from the tainted variable are also marked as tainted. We define two special sets for taint analysis: ${Func}_{taintsrc}$ which includes functions that are sources of contamination, and ${Func}_{taintsink}$ which includes functions where tainted parameters can lead to vulnerability points. A taint state τ is used to keep track of whether variables are tainted or not. The join operation ∨ returns “tainted” if at least one of the two variables being joined is tainted [33].

**Figure 5:**
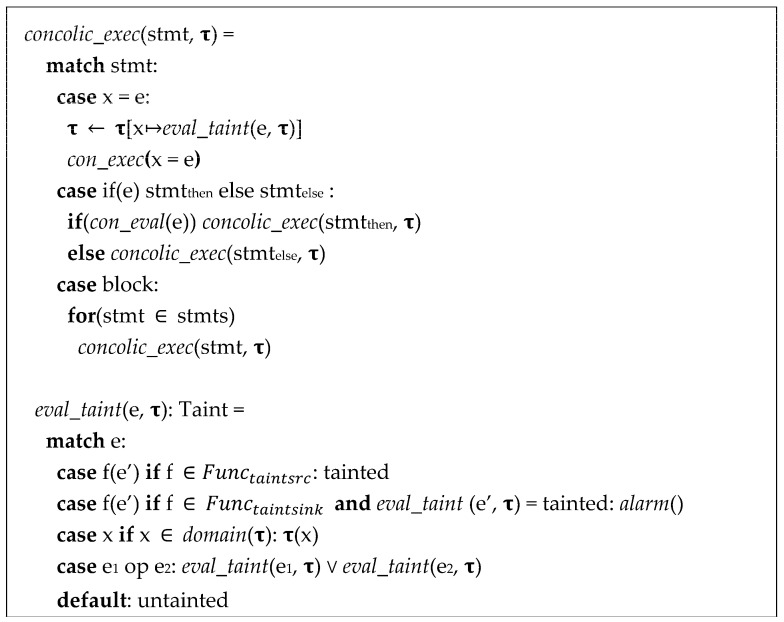
Semantics of concolic execution for dynamic taint analysis.

In Figure 5, our proposed scheme of dynamic taint analysis embedded into concolic execution semantics is presented. When executing the statement using stmt, it updates the taint state **τ** (con_exec). To evaluate the taint value of the expression e, the taint state **τ** is used. An assignment statement, such as x = e, renews the taint status of x by taint-evaluating e and updating x′s taint status as follows: **τ**[x ↦ eval_taint(e, **τ**)]. For evaluating a function f, it returns “tainted” if f is a taint source. In cases where f is a taint sink and the taint evaluation of the argument e′ is *tainted*, it triggers a taint alarm (alarm). If the expression corresponds to the variable x and it falls within the domain of the taint state **τ**, **τ**(x) is returned. When the expression involves operations between two expressions, the values obtained from taint-evaluating the two expressions are combined using the ∨ operator. For all other cases, it returns *untainted*. We will describe the taint analysis scheme together with concolic execution in more detail in Section 4.2.

### 3.3. Constraint Solving

Constraint solving is the process of determining the satisfiability of a set of constraints and, if satisfiable, finding solutions that satisfy those constraints. Typically, symbolic execution or concolic execution techniques choose and utilize a constraint solver that aligns with the modeling approach and analysis objectives [8]. During the symbolic execution, we use the path constraint *π* along with an SMT (Satisfiability Modulo Theory) solver to determine if the symbolic execution is feasible, to find models for the symbolic values (which represent the actual values of the program’s inputs), and to check if the assert statements inserted into the execution trace are satisfiable [34].

In our framework, we used Z3 as a constraint solver [35]. Our focal constraints encompass a range of four basic arithmetic operations (+, −, *,/) and string operations as permitted by the Z3 solver, including equations and inequalities. The string operations involve functions such as contains, concat, startwith, and others. Illustrative examples of these constraints include x.contains(“<”), y + 3 > 4, and concat(“alert”, z) = “alert()”.

In the context of path constraints, particularly when dealing with a “*π* = *π*′ ∧ e_s_”, our objective is to facilitate the exploration of unvisited branches in subsequent executions. To achieve this, we assess the satisfiability of *π*′ ∧ ¬ e_s_, where ¬ e_s_ signifies the negation of the symbolic expression e_s_, representing the condition expression of the most recently traversed branch. If this evaluation yields satisfiability, we derive a viable model denoted as I for utilization in the subsequent execution. In cases where satisfiability is not achieved, we proceed using *π*′ in a similar manner. If the symbolic expression of the path constraint *π* is satisfiable, then the execution path represented by the symbolic expressions is reachable by a certain combination of inputs, which is feasible. If the symbolic expression of the path constraint *π* is not satisfiable, meaning that there is no combination of inputs which satisfies the execution path represented by, that execution path is not realized.

We propose the integration of a concolic execution scheme with dynamic taint analysis. We use the dynamic taint analysis to address the significant limitations of static taint analysis, as mentioned earlier. We test the servlet code using multiple sets of inputs to maximize code coverage while keeping the test time as short as possible using concolic execution.

**Figure 6:**
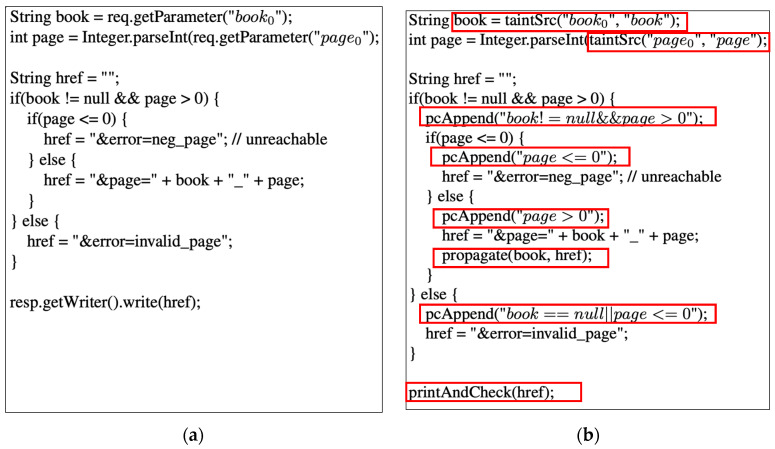
(**a**) A motivating example for Java Servlet Code. (**b**) Instrumented Servlet Code for dynamic taint analysis with concolic execution. All newly added codes are red-boxed.

Figure 6a shows a motivating example. There are two variables, book and page, which store user inputs. The subsequent if statements’ execution path is determined by the values of these user inputs. The string variable href, used as a parameter in the last statement res.getWriter().write(), is dependent on the user inputs. If the user input book is null, the else part of the outer if statement is executed. In this scenario, the variable href does not include untrusted user input, ensuring that the generated html is safe from XSS attack. However, if the user input book is not null and the page is greater than zero, the variable href may contain untrusted user input, making the generated HTML page vulnerable to XSS attack when passed to the statement resp.getWriter().write(). Therefore, the code is susceptible to XSS, and the challenge lies in detecting an XSS vulnerability that relies on the execution path.

## 4. Proposed Method

### 4.1. Extended Tomcat Servlet Runner for Concolic Execution

**Figure 7:**
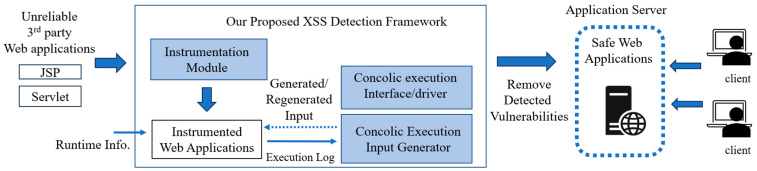
Execution and analysis flow of proposed XSS vulnerability detection framework on application server.

In Figure 7, we present the schematic view of our proposed framework for detecting XSS vulnerabilities in web applications. The primary objective of our framework is the automated identification of XSS threats via concolic executions, enabling web application server owners to mitigate the XSS security risk associated with third-party web applications. As presented in [32], to perform concolic execution, the executor requires support for two major functions: (1) an interface of input/output that enables repeated symbolic executions; (2) the tracer that ensures coverage of all execution paths. In a Java EE web application, a servlet handles a user’s HTTP request as an input and generates the HTTP response as the result. To implement our framework, we extend the Apache Tomcat Servlet runner [36] (version 9) to provide support for the concolic execution of the JAVA servlet code under test, which is either generated from JSP or handwritten. As shown in Figure 6, our framework executes instrumented web application code. During its execution, we enabled the automatic tracing of execution paths as well as dynamic taint analysis. The tracing of the execution path and taint analysis are achieved via the instrumentation of the servlet code. As a demonstration, the red boxes in Figure 6b are the instrumented code of the code shown in Figure 6a.

The execution path is dependent on the control flow. The path constraint is the execution trace log, which is the collection of the taken “branch conditions”. All the conditions of taken branches are collected in the form of symbolic expressions. The path constraint is sent to the concolic execution input generator, which includes the Z3 constraint solver. With its path constraint, the input generator finds the new input for the next iteration of concolic execution. As described in [19], the input generator enumerates all possible combinations of the listed branch conditions in the servlet code under test. It will generate the candidate conditions for the next round of iteration by negating subsets of branch conditions. If the candidate constraints are satisfiable in the constraint solver, the input generator produces concrete input values that align with the candidate constraints. If the candidate constraints are unsatisfiable, it will try the satisfiability test of untested candidate constraints instead of generating concrete values.

The extended Tomcat interface is utilized to carry out the subsequent iteration of concolic execution. Using this interface, the model of candidate constraints which is generated by the input generator is translated into concrete values. The generation of new inputs and subsequent servlet execution will be iterated until all reachable execution paths have been covered. During the concolic executions, the input generator will maintain a log of tested candidate conditions. This approach effectively prevents redundant executions of identical execution paths, thus addressing the issue of execution explosion commonly faced in various dynamic analyses.

### 4.2. Instrumentation Module

To generate an execution log, it is necessary to accumulate all branch conditions that are encountered during the execution of the servlet. Simultaneously, to facilitate dynamic taint analysis, the flow of taint information needs to be traced. To address these requirements, we instrument the Java servlet code. The instrumentation of the servlet code involves inserting and/or replacing some methods with new ones both for dynamic taint analysis and concolic execution.

We detect an XSS threat using dynamic taint analysis along with concolic execution. When the instrumented code is running on an extended servlet runner, all tainted variables are collected via the explored execution path. In every concolic execution, taint information is propagated from taint source to taint sink. We have built the taint propagation analysis, which performs information flow analysis starting from taint source to taint sink. We define the vulnerability and the taint propagation as follows:

- User inputs (including cookie and run-time settings) are tainted when analysis starts;
- The left-hand-side variable of the assignment statement is tainted if the right-hand side contains tainted variable(s);
- The variable copied from the function call and return value are tainted if the source is tainted;
- If the tainted variable is used at the taint sink, it is vulnerable.

Considering user input as potentially tainted, it is necessary to tag all input data as tainted and record attributes, such as types and names. For each input receiving variable, we mark them as “tainted”. Since the taintedness of one variable will be propagated to other variables during execution, we need to trace the “usage” of tainted variables. Therefore, we need to update the list of tainted variables in assignment statements and function calls with parameters. In the taint sink, we need to verify whether the tainted variable is used to generate output HTML or not. In our proposed instrumentation module, we add these functionalities into the servlet code in regards to the dynamic taint analysis.

For concolic execution, there are steps to follow. Initially, the executed control paths are traced with provided initial inputs. Then, new inputs are generated to execute unexplored execution paths. This process is repeated until all execution paths are explored. Through these steps, we trace all branch conditions of the servlet code. Our instrumentation module performs the following functionalities:

- Records user inputs that influence the control flow as symbolic values;
- Updates the variable’s symbolic expression when a variable is updated on change of user-input and performs the symbolic evaluation;
- Represents the changes in the control flow of the execution path (such as branch, loop statements, function calls, etc.) as the symbolic expression for regeneration of input data.

To enable concolic execution with dynamic taint analysis, we implemented four methods. The roles of these methods are listed in Table 2.

TaintSrc is a method used for input variables. It translates concrete input values into symbolic values for concolic executions and marks input variables as tainted for dynamic taint analysis. The input receiving methods such as getParameter or getAttribute in Figure 6a are replaced with the TaintSrc method as in Figure 6b.

The Propagate method updates the symbolic values of variables and keeps the flow of taintedness. The assignment statement and function calls generate the transition of taintedness from one variable to another. To trace the transitions, we insert the Propagate method into the assignment statements with tainted variables. We also add this method to function calls whose parameters are tainted or return values that are tainted with a function call.

In the taint sink, it is necessary to verify whether there is a potential vulnerability threat in the servlet code execution arising from the utilization of a tainted variable. By substituting the original HTML-generating method with instrumented printAndcheck, we can effectively identify the potential security risks.

We insert the trace logging method into the control dependent code segment, especially focusing on the symbolic expressions of input. For example, as shown in Figure 6, the method PcAppend(), which generates symbolic expressions of branch condition expressions, is inserted into the very beginning of both *then* basic blocks and *else* basic blocks. The logs of this statement are symbolic expressions of branch conditions.

### 4.3. New Input Generation and Code Coverage

We implemented an input generator which enabled the repeated execution of concolic execution by generating new inputs. It generates new input data that guarantees execution of the previously unexplored execution paths and repeatedly executes the servlet code until all execution paths are explored. Our input generator processes two essential data to support this scheme. The execution trace log, the path constraint, that represents the conditions of explored execution paths, and the input map, which is the history of generated inputs. In the input generator, we employ the Microsoft Z3 solver [35] as our SMT (Satisfiability Modulo Theories) solver, which can handle string logic formulas with logical operators.

The execution path is the sequence of explored basic blocks when specific inputs are given, and the path constraint is represented as symbolic expressions of conditions for changing the control flows. As explained in Section 3.3, the path constraint is the logical formulas of symbolic expressions, which contain arithmetic operators or string operators. The path constraint consists of the sequence of symbolic expressions of the conditional statements; therefore, the generated path constraint represents one executed concolic execution flow. Since the PcAppend method, which generates the symbolic expression of control flow, is inserted into the next statement of the branch instruction, the length of the symbolic expressions of the generated trace log is the number of executed conditional statements on the code. Therefore, there are a maximum of 2^n^ different combinations of execution flows, where the number of conditional statements in the program is n.

Whenever the servlet terminates one iteration of concolic execution, we can find the input model for the next iterations from the path constraint. To find the new input for the unexplored execution path, we need to find the set of inputs which represents symbolic expressions which have not been explored. We can find the symbolic expressions of the candidate input model by negating the sub-conjunct of the executed path constraint.

By checking the satisfiability of the symbolic expressions of the candidate input using the Z3 solver, we can identify whether the input model is feasible or not. If a satisfiable model exists, the input map is updated with this model so that it can be used as the input for the next execution. If it is not satisfiable, the last conjunct is removed, and the same process is repeated for the remaining conjuncts in the path constraint. In this process, the new input should execute the different execution path. When the path constraint list is exhausted, the constraint solving is stopped and no additional execution is required. As with other concolic execution methods, we use a depth-first search strategy for path exploration. To prevent the duplication of previously visited execution paths, we maintain the input map, the information about which branch each conjunct of the path constraint was created from. Additionally, the attempt to collect path constraints in loops is limited to k-times (a given constant) to limit the number of path constraints collected.

## 5. Applications and Experiments

We implemented Instrumentation Module (IM) to inject codes and replace taint-related methods. Our IM is implemented based on the static compile time analyzer using ASM library [37] version 8.0, which is a JAVA bytecode manipulation framework.

### 5.1. Applications

**Figure 8:**
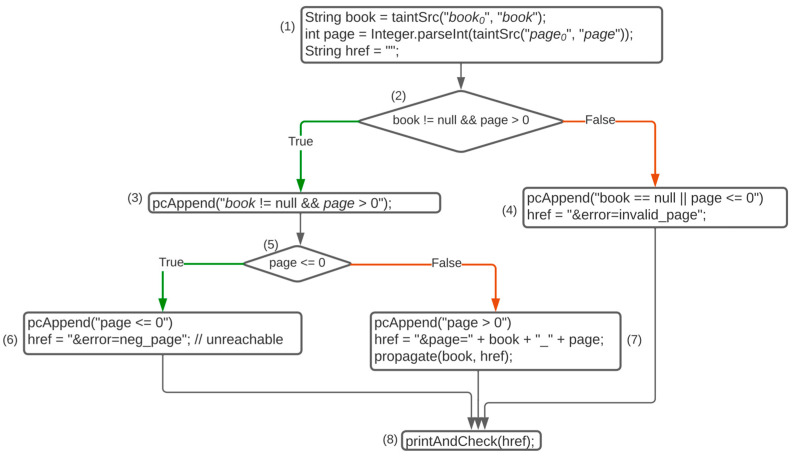
Control flow of dynamic taint analysis.

Figure 8 shows the instrumented code’s control flow and taint propagation under concolic execution of the motivating example, and Table 3 shows the information stored for dynamic taint analysis under concolic execution of the motivating example. The numbers (1)–(8) are the basic blocks. While it is executed, the taint analysis information is generated for all taint-related variables from taint source to taint sink. At the same time, all execution flows and path constraints are gathered at the level of basic block. In the initial concolic execution, randomly generated input is given. As the branch conditions are concretely fixed, the execution flow is generated as the sequence of basic blocks.

In the initial concolic execution, suppose that inputs *a* and 10 are given for book_0_ and page_0_. As the first row of Table 3, the first branch condition is true, and the second branch condition is false; therefore, the execution path is (1) (2) (3) (5) (7) (8). The symbolic value of book and page are stored as book_0_ and page_0_ in basic block (1) by the taintSrc method, where both are tainted. The statements “String id = taintSrc(“book_0_”)” and “int page = integer.parseInt(taintSrc(”page_0_”)” propagate taintedness from taintSrc(“book_0_”) to book and taintSrc(”page_0_”) to page. In block (2), the condition of the first if statement, (book == null && page > 0), is true and the instrumented code pcAppend(book == null && page > 0) in (3) accumulates the symbolic expression “*book*_0_ *! = null && page*_0_ *> 0*” into the path constraints. Since the condition of the second if statement in block (5) is false, “*page > 0*”, the negation of condition, is accumulated in the path constraints in block (7) by the pcAppend method. Finally, the executed path constraint of this initial execution, let pc_0_, is (*book*_0_* ! = null && page*_0_* > 0)* ∧ *(page*_0_* > 0)*. For the dynamic taint analysis, this code is vulnerable because the variable “href”, which is tainted from book via href, is used in printAndCheck(href).

Following the initial execution, the input for the next iteration should be generated. As described in Section 3.3, the symbolic expressions of path constraints are the log of the traversed execution path. To evaluate taintedness in all feasible execution paths, we need to generate input values that correspond to the entire spectrum of symbolic expressions representing execution paths. To execute via unexplored execution paths, it is necessary to generate input values that correspond to symbolic expressions not previously generated. To find such concrete inputs, we negate certain sub-expressions of the symbolic expressions of previously generated execution paths.

To execute unexplored paths, it is necessary to derive the concrete values of the symbolic variable, book, and page that satisfy the negated last component, (page_0_ *≤* 0), of the path constraint collected in the initial execution. The new path constraint, denoted as pc_1_ for the next iteration, becomes (*book*_0_ *! = null && page*_0_ *> 0)* ∧ *(page*_0_ *≤ 0*). However, this symbolic expression is not satisfiable, which means that the concrete values that satisfy the symbolic expression do not exist, and, consequently, pc_1_ cannot be the possible execution path.

As demonstrated in the second row of Table 3, the symbolic expression for the next iteration of concolic execution becomes *(book*_0_ *! = null && page*_0_ *> 0),* which is satisfiable, and we can generate the concrete input for the next iteration. With these satisfiable concrete values of input for book and page, such as x and 0, the execution flow becomes (1) (2) (4) (8). Since href does not have a tainted symbolic expression in this execution path, it is not vulnerable when the statement printAndCheck(href) checks taintedness. In this specific example, all feasible execution paths are completely covered within two iterations of concolic execution.

### 5.2. Experimental Results

We experimented using SecuriBench Micro version 1.08 [38] and OWASP v1.2 [39]. The Tomcat (version 9) servlet runner is extended in its input/output interface to handle concolic executions. The interface can decide whether the concolic execution should be repeat with newly generated input or stop. The instrumentation (described in Section 4) is applied into the servlet-generation class by using JVM TI (Java Virtual Machine Tool Interface). The benchmark analysis is performed on a system with an Intel (Santa Clara, CA, USA) Core i5 2.3 GHz CPU and 16 GB of physical memory running on Apple (Cupertino, CA, USA) macOS Monterey with Oracle (Austin, TX, USA) Java Runtime version 1.8 (64 bit). The instrumentation and analysis phases are processed offline, which means that our proposed method should find XSS vulnerabilities before the initiation of the web application server to detect XSS vulnerability.

SecuriBench Micro is a suite of J2EE micro benchmarks originally intended for web-based applications. It has 123 test files grouped into 12 categories. The current version of our scheme can handle six categories of test cases: aliasing, arrays, basic, pred, session, and strong updates. Each test case is a self-contained servlet. We tested 75 test files of XSS threats. Considering the diversities of tested cases in the benchmark, we believe that it is enough tests to validate our proposed approach for the detection of XSS threats.

Table 4 shows our experimental results. In each test case in Securibench, “bad”, “ok”, and “etc” are marked in the code. “Bad” means an actual vulnerability that the analyzer should detect. “Ok” means a fake vulnerability that the analyzer should not detect (false alarm). According to the results, our scheme detects most of the true vulnerabilities, with a TPR (True Positive Ratio) of 93.75% in the target test cases and without any false positives, with a 0% FPR (False Positive Ratio).

Among Securibench benchmark suites, we excluded some test cases, categorized as data structures, factories, inters, reflection, and sanitization, from the experiments. Currently, those categories of tests cannot be handled properly because of dynamic data structures or randomized inputs which are unable to generate symbolic values. Test cases including SQL injection and http splitting attacks are not tested, because those threats are not within the scope of this research.

The OWASP (Open Web Application Security Project) benchmark offers a set of vulnerability test cases to evaluate various Application Security Testing tools. In Version 1.2, it provides a total of 2740 test cases for vulnerabilities, including command injection, weak cryptography, weak hashing, LDAP injection, path traversal, secure cookie flag, SQL injection, trust boundary violation, weak randomness, XPath injection, and Cross-Site Scripting (XSS). Among these, the main focus of this paper, XSS, is covered by 455 test cases, with 246 of them containing actual vulnerabilities and 209 false vulnerabilities to verify tool detection accuracy.

Table 5 shows the results using a total of 455 test cases, where 170 are True Positives (TP) and 76 are False Negatives (FN), indicating that the tool detects a significant portion of the 246 cases that it should. There are 209 True Negatives (TN) and 0 False Positives (FP), meaning the tool correctly refrains from detecting all 209 test cases where it should not. Our scheme exhibits a TPR of 69.11% and an FPR of 0%. It is important to note that this is the initial prototype version, and the true positive rate is expected to increase in the future as we expand grammar coverage and supported data structures.

**Figure 9:**
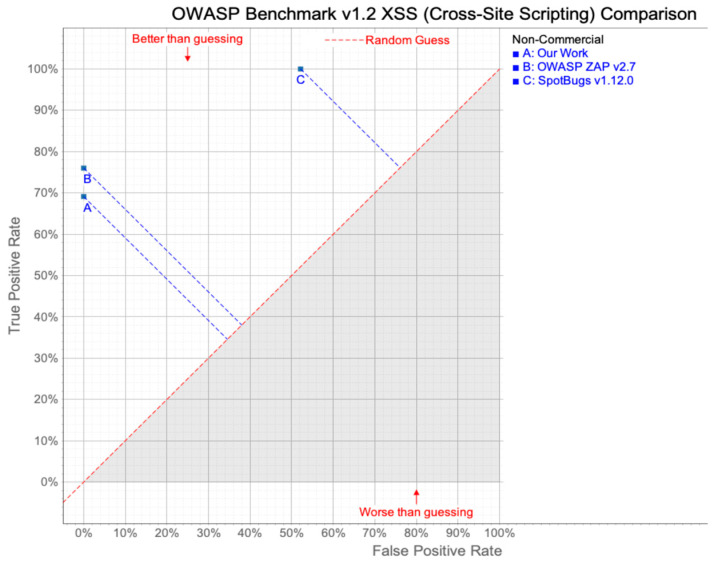
OWASP Benchmark v1.2 XSS (Cross-site Scripting) comparison using perf comparison Adopted with permission from Ref. [39]. Copyright 2007, Free Software Foundation. The grey colored area stands for the FPR is greater than TPR, which is worse than the “random guess”.

Figure 9 compares application security testing tools using OWASP benchmark test cases related to XSS [39]. Using these tools, we can compare the performance of vulnerability detection by visualizing TPR and FPR. In this research, we compared two famous vulnerability detection tools with ours. The static analysis tool SpotBugs, when equipped with Find Security Bugs, demonstrates a high TPR of 100% but also exhibits a relatively high FPR exceeding 50% (point C in Figure 9). It has the drawback of including a notable number of false positives [21]. In the case of ZAP [24], it demonstrates superiority over the results presented in this paper for XSS targets in the OWASP Benchmark, achieving a TPR of 76% and an FPR of 0% (point B in Figure 9). However, it is noteworthy that ZAP requires crawling the target web application to generate test cases. While a higher number of test cases generally provides broader coverage, an increased test case count may also lead to the execution of duplicate execution paths, resulting in longer testing durations. According to [24], using ZAP version 2.7, they reported an average time expenditure of approximately 6.5 h.

In contrast, our tool presents a TPR close to 70% and a 0% FPR. This suggests that our tool performs favorably, with no false alarms and a high level of actual vulnerability detection. We anticipate achieving an even higher TPR in the future by expanding the supported grammar and data structures. For the speed of analysis, it took 57 s to analyze a total of 11,925 lines of code. This is approximately 209 locs/sec. We believe this analysis speed is reasonable for real-world applications.

## 6. Conclusions and Discussion

The prevention of vulnerability to XSS of a web application is a very important issue for enhancing the security of the IoT application layer. To address this issue, we proposed a dynamic taint analysis scheme using concolic execution for Java EE platforms. Our proposed scheme exhibits no false alarms while removing unnecessary repeated executions of dynamic taint analysis, thereby optimizing the efficiency of dynamic taint analysis.

In this work, we have developed a framework for web application runners which can “concolically” execute the code under test. We defined an instrumentation scheme to handle taint analysis and concolic executions and successfully automated the instrumentation process for the Java servlet code and JSP. This approach allows us to precisely identify the input dataset responsible for bringing about XSS threats. Our experimental results on partial test sets of SecuriBench Micro and OWASP benchmark demonstrate the validity of the proposed scheme. Our method achieved a detection rate (true positive) of 90.62% of XSS threats using Securibench and 69.11% using OWASP while showing 0% false positives.

Furthermore, we compared the performance of our framework with conventionally available XSS detection tools, including two static taint analysis tools and one dynamic taint analysis tool using OWASP benchmark. The results reveal that our proposed method successfully avoids the “misdetection” of the false-positive XSS vulnerabilities, a common drawback of the static taint analysis schemes. In contrast to the conventional dynamic taint analysis, we argue that the analysis speed facilitated by concolic executions dramatically reduces the analysis time while maintaining a reasonable TPR. Our framework presents a practical and effective solution for web application developers and security practitioners, offering a means to identify and address XSS vulnerabilities in web application services.

The current version of implementation has some limitations. Firstly, it has the inability to analyze code that contains complex data structures. Secondly, we only handle XSS detection in JSP or Java Servlet Code. As a part of our future work, we aim to enhance the framework by extending its capabilities to encompass complex data structures and a wider spectrum of taint-related vulnerabilities. Furthermore, we intend to strengthen our framework by adding XSS detection capabilities in JavaScript. We plan to improve our analysis techniques to handle malicious code injection attacks in the application layer, malicious code injection attacks in the perception layer, and SQL injection attacks in the support layer. This will ultimately enhance the security of IoT.

## Figures and Tables

**Table 1:** Roles of four layers of IoT and representative security attacks [2].

Layer	Roles	Representative Security Attacks
Perception Layer	Collect raw data using sensors, actuators, and controllers	Node Capture Attack, Malicious Code Injection Attack, False Data Injection Attack, Side Channel Attack
Network Layer	Transport data via networks (BLE, Wifi, 5G, etc.)	Phishing attack, DDoS attack, Data Transit Attack, Routing attack, Storage Attack
Support Layer	System-level data processing management (e.g., computing, resource, storage management)	DDoS Attack, Man-in-The-Middle Attack, SQL-injection
Application Layer	Interfaces with end-users Provides diverse web-based services	Cross-site Scripting, Access control attack, Malicious Code Injections, Sniffing

**Table 2:** The instrumentation policy and the roles of instrumented methods.

Method	Parameters	Instrumentation Policy	Roles
TaintSrc	user input	Replace function f with TaintSrc if $f\in {Func}_{taintsrc}$	Handle user input as tainted (DTA **) Generate symbolic expression of user input (CE ***)
Propagate	tainted variable	Add after assignment statement if tainted variable is used	Trace the propagation of tainted variables (DTA) Update symbolic expressions of variables (CE)
PrintAndCheck	http output	Replace function f with PrintAndCheck if $f\in {Func}_{taintsink}$	Check the taintedness of argument expression and output html (DTA)
PcAppend	branch condition (string)	Add *e_s_* * and ¬*e_s_* at the head of ${stmt}_{then}$ and ${stmt}_{else}$ for $if\left(e\right)\;{stmt}_{then}\;else\;{stmt}_{else}$	Conditions of branch are accumulated to trace log (CE)

* *e_s_* is symbolic expression of e; ** DTA indicates dynamic taint analysis; *** CE indicates concolic executions.

**Table 3:** Symbolic values and execution paths of two iterations of concolic execution.

Variables					Execution Path	Path Constraint
book (conc.)	page (conc.)	book (symb.)	page (symb.)	href (symb.)		
“a”	10	book_0_	page_0_	“&book =” + book_0_ + “_” +page_0_	(1)(2)(3)(5)(7)(8)	(*book*_0_ *! = null && page*_0_ *> 0)* ∧ *(page*_0_ *> 0)*
“x”	0	book_0_	page_0_	“&error = invalid_page”	(1)(2)(4)(8)	(*book*_0_ *== null || page_0_ ≤ 0)*

**Table 4:** Taint analysis results of XSS using Securibench Micro v1.08.

Categorical Sets	XSS Detection Results	
	TP/(TP + FN)	FP/(TN + FP)
Aliasing	10/10	0/2
Arrays	9/9	0/6
Basic	33/37	0/6
Pred	3/5	0/4
Session	3/3	0/1
Strong_updates	0/0	0/4
Total	58/64 (TPR 90.62%)	0/23 (FPR 0%)

**Table 5:** Taint analysis results of XSS using OWASP Benchmark v1.2.

XSS Detection Results						
TP	FN	TN	FP	Total	TPR	FPR
170	76	209	0	455	69.11%	0%

## Data Availability

Data are contained within the article.
